# Photo‐Chemical Stimulation of Neurons with Organic Semiconductors

**DOI:** 10.1002/advs.202300473

**Published:** 2023-09-03

**Authors:** Achilleas Savva, Adel Hama, Gabriel Herrera‐López, Tony Schmidt, Ludovico Migliaccio, Nadia Steiner, Malak Kawan, Hubert Fiumelli, Pierre J. Magistretti, Iain McCulloch, Derya Baran, Nicola Gasparini, Rainer Schindl, Eric D. Głowacki, Sahika Inal

**Affiliations:** ^1^ Biological and Environmental Science and Engineering King Abdullah University of Science and Technology (KAUST) Thuwal 23955‐6900 Saudi Arabia; ^2^ Department of Chemical Engineering and Biotechnology University of Cambridge Cambridge CB30AS UK; ^3^ Gottfried Schatz Research Center Chair of Biophysics Medical University of Graz Neue Stiftingtalstraße 6 Graz 8010 Austria; ^4^ Bioelectronics Materials and Devices Laboratory Central European Institute of Technology Brno University of Technology Purkyňova 123 Brno 61200 Czech Republic; ^5^ Physical Science and Engineering (PSE) KAUST Solar Center (KSC) King Abdullah University of Science and Technology (KAUST) Thuwal 23955‐6900 Saudi Arabia; ^6^ Department of Chemistry and Centre for Processable Electronics Imperial College London London W12 0BZ UK

**Keywords:** non‐fullerene acceptors, organic bioelectronics, photo‐stimulation

## Abstract

Recent advances in light‐responsive materials enabled the development of devices that can wirelessly activate tissue with light. Here it is shown that solution‐processed organic heterojunctions can stimulate the activity of primary neurons at low intensities of light via photochemical reactions. The p‐type semiconducting polymer PDCBT and the n‐type semiconducting small molecule ITIC (a non‐fullerene acceptor) are coated on glass supports, forming a *p*–*n* junction with high photosensitivity. Patch clamp measurements show that low‐intensity white light is converted into a cue that triggers action potentials in primary cortical neurons. The study shows that neat organic semiconducting *p*–*n* bilayers can exchange photogenerated charges with oxygen and other chemical compounds in cell culture conditions. Through several controlled experimental conditions, photo‐capacitive, photo‐thermal, and direct hydrogen peroxide effects on neural function are excluded, with photochemical delivery being the possible mechanism. The profound advantages of low‐intensity photo‐chemical intervention with neuron electrophysiology pave the way for developing wireless light‐based therapy based on emerging organic semiconductors.

## Graphical abstract

Highly photosensitive, organic *p*–*n* junctions stimulate primary neurons via photochemical reactions, when illuminated with low‐intensity light. The profound advantages of low‐intensity, photo‐chemical intervention with neuron electrophysiology, pave the way for developing wireless, light therapy, based on emerging organic semiconductors.

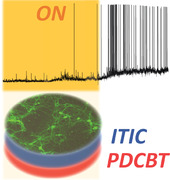

## 1 Introduction

Biomedical engineering concepts that use light to control cellular activity have been widely used in clinical practice in different medical fields, such as oncology^[^ ^1^ ^]^ and ophthalmology.^[^ ^2^ ^]^ Recent advances in light‐responsive materials, unlock new concepts, spanning from artificial vision^[^ ^3^ ^]^ to wireless stimulation of the nervous system,^[^ ^4^ ^]^ as well as tissue regeneration via phototherapy.^[^ ^5^ ^]^ In general, using exogenous functional materials to selectively manipulate cell activity is considered a less risky approach compared to optogenetics.^[^ ^6^ ^]^ Biology‐guided design principles have established intracellular and extracellular material interfaces able to photo‐stimulate a range of biological systems. Successful examples include carbon‐based nanomaterials^[^ ^7^ ^]^ and gold nanoparticles^[^ ^8^ ^]^ for photo‐thermal cancer treatment, silicon nanowires for photo‐modulation of neurons,^[^ ^9^ ^]^ and other light‐responsive silicon‐based structures to control biological systems.^[^ ^10^ ^]^

Recently, organic photo‐actuators—devices that use organic semiconductors able to modulate cell activity with light—have attracted significant interest because of relatively simple fabrication requirements and device setup,^[^ ^11^ ^]^ biocompatibility, and strong absorption of light in the visible spectrum (i.e., wavelengths spanning from ≈400 to 700 nm). For in vitro applications, organic semiconductor thin films are deposited on transparent conducting substrates and directly interfaced with cells that are cultured on their surface. Light can be simply absorbed by the photosensitive organic semiconductor and converted into a cue that modulates the activity of cells in close proximity. These unique advantages have been leveraged to build organic photo‐actuators with high temporal and spatial resolution for both in vitro^[^ ^12^ ^]^ and in vivo^[^ ^13^, ^14^ ^]^ applications. Other than neurons, photo‐actuators were also proven successful in controlling the behavior of non‐electrogenic cells, such as astrocytes,^[^ ^15^ ^]^ endothelial colony‐forming cells,^[^ ^16^ ^]^ and embryonic kidney cells (HEK‐293) with light.^[^ ^17^ ^]^ Other applications of organic photo‐actuators include light‐mediated protein modification,^[^ ^18^ ^]^ controlled delivery of reactive oxygen species in cell cultures,^[^ ^19^ ^]^ and modulation of redox equilibrium in model organisms.^[^ ^20^ ^]^

The mechanisms of controlling the activity of biological systems with organic photo‐actuators can be quite diverse and are still not well understood.^[^ ^21^ ^]^ In general, it is suggested that organic semiconductors stimulate cells upon light exposure via three major mechanisms, i.e., photo‐thermal,^[^ ^12^ ^]^ photo‐capacitive,^[^ ^22^ ^]^ and photo‐electro‐chemical^[^ ^16^ ^]^ stimulation. Photo‐thermal stimulation usually occurs when photo‐generated excitons in organic semiconductors—a bound hole/electron pair^[^ ^23^ ^]^—recombine, releasing their energy as thermal radiation. This mode of operation usually occurs when a single organic semiconductor film is used as the photosensitive component. The photosensitive component can also be made from a combination of p‐type (hole transporting) and n‐type (electron transporting) organic semiconductors, to form light‐sensitive *p*–*n* junctions.^[^ ^24^ ^]^ These *p*–*n* junctions are generally able to deliver large photovoltage and photocurrent at the polymer/cell interface due to exciton splitting that minimizes charge recombination. As a result, free electrons interact with cations in the electrolyte and induce a photo‐capacitive modulation of the cell membrane.^[^ ^25^ ^]^ Free electrons can also react with oxygen or other electrolyte species to form reactive oxygen species (ROS) and hydrogen peroxide (H_2_O_2_). These photo‐faradaic reaction products can trigger ion channels on the cell membrane to modulate cell behavior. Overall, the photo‐stimulation mechanisms involved in organic photo‐actuators can be complex^[^ ^26^ ^]^ and specific for different biological systems and materials used.

Organic semiconductors can be molecularly tuned by chemical synthesis to achieve unique absorption profiles, leading to photo‐actuators with sensitivity to specific wavelengths^[^ ^27^ ^]^ and intensities.^[^ ^28^ ^]^ For instance, Głowacki group developed organic *p*–*n* junctions based on conjugated small molecules and used them for the photo‐capacitive stimulation of neuronal cultures^[^ ^24^, ^29^ ^]^ and single cells (i.e., oocytes).^[^ ^30^ ^]^ These devices are sensitive in the deep‐red region of the optical spectrum (i.e., ≈650 nm) and can photo‐stimulate cells at light intensities in the range of 100–1000 mW cm^−2^. Earlier studies from Lanzani and co‐workers used the p‐type polymer poly(3‐hexylthiophene) (P3HT) and the n‐type small molecule^[^ ^6^ ^]^ phenyl‐C61‐butyric acid methyl ester (PCBM) to trigger action potentials in primary hippocampal neurons upon light illumination.^[^ ^31^ ^]^ These results revealed the potential of organic semiconductors for photo‐stimulation of a wide range of biological systems. However, several p‐type and n‐type organic semiconductors with significantly improved optoelectronic properties have been recently developed for solid‐state optoelectronic applications.^[^ ^32^ ^]^ In particular, non‐fullerene electron acceptors have revolutionized the field of organic solar cells due to their excellent charge transport, strong optical absorption in the visible and near‐infrared region, and better energetic match with the donor material.^[^ ^32^ ^]^ These unique features can open new horizons for organic photo‐actuators and potentially enable the photo‐stimulation of biological systems at ultra‐low light intensities.

Here we developed an organic bilayer *p*–*n* junction based on the p‐type polymer poly[2,2‴′ – bis [[(2‐butyloctyl)oxy]carbonyl] [2,2′:5′,2″:5″,2‴‐quaterthiophene] −5,5‴‐diyl] (PDCBT) and the n‐type small molecule 3,9‐bis(2‐methylene‐(3‐(1,1‐dicyanomethylene)‐indanone))−5,5,11,11‐ tetrakis (4‐hexylphenyl) – dithieno [2,3‐d:2′,3′‐d″]‐s‐indaceno[1,2‐b:5,6‐b″] dithiophene (ITIC). By activating this *p*–*n* bilayer junction coated on glass coverslips with light, we could control the active electrophysiology of primary (mouse) cortical neurons without any conducting substrates or external electrodes. Despite that we focused on neat organic semiconducting films, we first studied these bilayer junctions on a photodiode setup by spin‐coating the material solutions on a glass/Indium‐tin oxide (ITO) substrate, forming homogeneous PDCBT/ITIC bilayers with an overall thickness of 55 nm. The *p*–*n* bilayer stack absorbed visible light of a wide range of wavelengths and exhibited excellent operational stability in aqueous electrolytes, as verified by electrochemical quartz crystal microbalance studies *in operando*. Photo‐electrochemical measurements revealed that high photovoltage (>0.3 V) and high photocurrent (>0.6 mA cm^−2^) values were produced when the device was illuminated with low light intensity (≈40 mW cm^−2^). These results reveal the potential of these emerging materials to be used in established photo‐stimulation devices for highly sensitive wireless control of neuronal activity. The biocompatibility of these materials was evaluated by live/dead assays of different cell lines (MDCKII, HEK293, and primary neurons) cultured directly on the film's surface. Several individual neurons were probed with the patch clamp method, and frequent action potentials were recorded when the device was illuminated with 40 mW cm^−2^ white light. Identically cultured neurons on control glass or glass/photoresist layers showed no action potential generation under the same illumination conditions, suggesting a non‐photo thermal activation pathway. Direct H_2_O_2_ stimulation is also excluded by monitoring the membrane potential of neurons grown on glass and on PDCBT‐ITIC coated glass substrates, while were perfused with H_2_O_2_ solutions of different concentrations (1 µm–10 mm). Controlled patch clamp measurements on neurons grown on partially covered glass slides—i.e., “half‐coated” with PDCBT‐ITIC—suggest photo‐generated electron transfer to oxygen and other chemical species present in the cell culture media, which most possibly are responsible for altering neuron electrophysiological activity. Possible photo‐chemical mechanisms are discussed as alternative pathways to wirelessly intervene with cell activity using light mediated by organic semiconductors.

## 2 Results and Discussion

**Figure 1:**
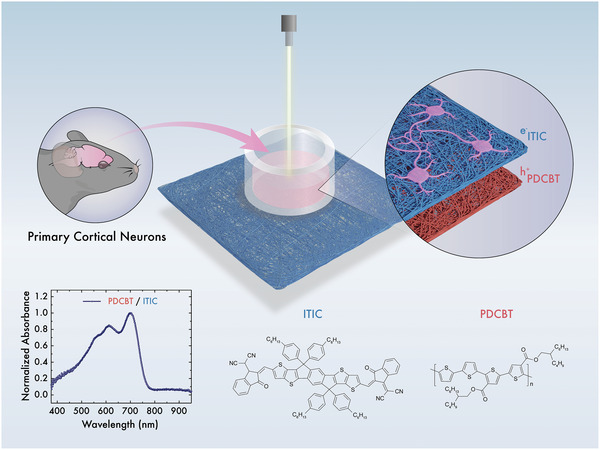
Schematic representation of the photo‐stimulation of neurons in vitro using an organic *p*–*n* heterojunction: PDCBT‐ITIC. Mouse cortical neurons were extracted and cultured directly on the photosensitive *p*–*n* polymer junction generated by layer‐by‐layer type spin coating of each film. The absorption spectrum of the *p*–*n* junction and the chemical structures of the semiconducting materials used are shown in the bottom panel.

Constructing an efficient and stable *p*–*n* junction photodiode using a layer‐by‐layer solution‐based process is challenging. Since these devices will operate in an aqueous environment, two main film‐processing conditions must be carefully considered for device stability: the solvent of choice to process the films and the adhesion of the *p*–*n* layers. To address these challenges, we first spin‐coated the p‐type polymer PDCBT from a chlorobenzene solution, followed by spin‐coating the n‐type non‐fullerene acceptor (i.e., ITIC) from a toluene solution. The orthogonal solvent of the n‐type counterpart ensures that the n‐type film casting does not damage the PDCBT film underneath. The chemical structures of the materials used and the absorption profile of the PDCBT‐ITIC *p*–*n* junction are shown in **Figure** **1**. Importantly, the optical absorption maximum of the proposed *p*–*n* junctions is at ≈720 nm, which is in line with the optimum phototherapeutic window.^[^ ^33^ ^]^ The cortical neurons used in this study were extracted from mice embryos and cultured on the surface of these films.

**Figure 2:**
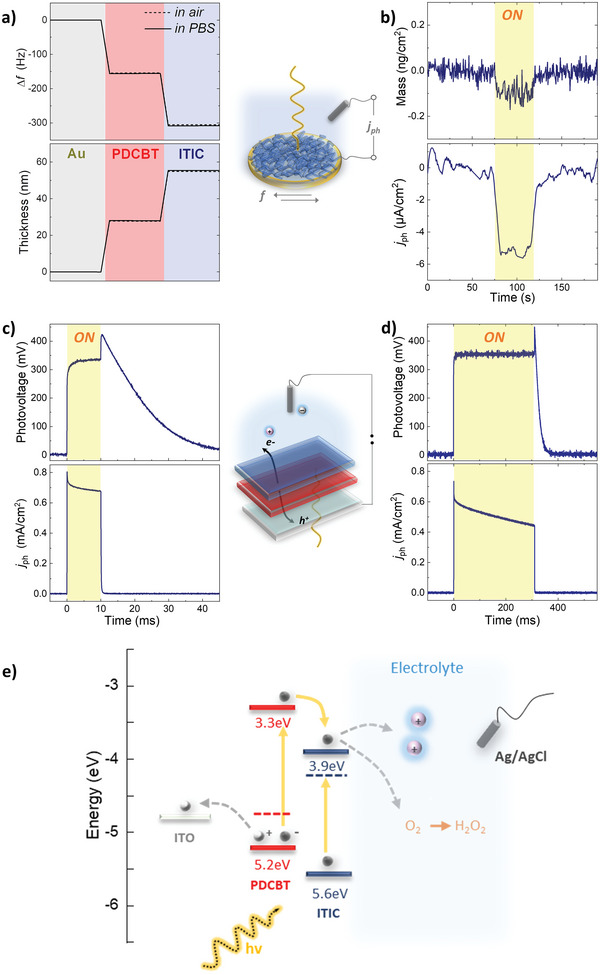
a) A schematic of the photo‐EQCM‐D setup, used to record mass and photocurrent changes *in‐operando*. The thickness of the PDCBT‐ITIC films was calculated using the QCM‐D data (top graph) in dry conditions (solid line) and when they were immersed in PBS (dashed line). b) Mass changes of the PDCBT‐ITIC films and the corresponding photo‐current changes upon illumination. c) A schematic of the photodiode setup used to study the photo‐electrochemical operation of the PDCBT‐ITIC junctions in aqueous electrolytes. The photovoltage and photocurrent density response of the system is shown when the stimulation is conducted with a red light pulse (*λ*_630nm_) with an intensity of 33 mW cm^−2^ for 10 ms and d) for 300 ms. e) An energy diagram showing the photodiode components and the photo‐electrochemical processes occurring upon light exposure, depicting photo‐capacitive charging at the semiconductor/electrolyte interface and photocathodic charge transfer to oxygen, producing hydrogen peroxide.

To understand the operation of our proposed *p*–*n* junctions in biologically relevant conditions, we performed a series of photo‐electrochemical measurements, which are summarized in **Figure** **2**. First, we used electrochemical quartz crystal microbalance with dissipation monitoring (EQCM‐D) equipped with a special module (Figure 2a,b) where window access allows for the simultaneous monitoring of mass and current changes upon illumination in operando. We first coated the PDCBT polymer film directly on the QCM‐D gold sensor and measured the film thickness in both dry conditions and phosphate‐buffered saline (PBS) solution, i.e., 27 and 28 nm, respectively (Figure 2a). We then spin‐coated the ITIC film on the same sensor, and its thickness was found to be 28 nm and 30 nm in dry and wet conditions, respectively. The overall thickness of the *p*–*n* junction remains almost unchanged when immersed in PBS for several hours (i.e., 55 nm in dry conditions and 58 nm in PBS), indicating a stable “wet” condition of these *p*–*n* junctions—a major requirement to interface them with biological systems. After the *p*–*n* junction was stabilized in PBS, a low‐intensity white light illumination was applied, and the photocurrent response, together with the *in‐operando* mass change of the polymer films, was recorded (Figure 2b). Upon illumination, an average photocurrent of ≈5 µA was recorded, accompanied by almost no change in the mass of these films. This observation indicates a photo‐electrochemical reaction at the organic semiconductor/electrolyte interface and opposes an ion‐uptake (or release) process, which would manifest as a mass change.^[^ ^34^ ^]^

We performed photo‐electrochemical measurements with controlled light intensity and pulse width to evaluate the magnitude of photocapacitive and/or photofaradaic currents, which the *p*–*n* junctions can produce. PDCBT and ITIC films were spin‐coated on ITO substrates and immersed in PBS with an Ag/AgCl electrode as the counter electrode (Figure 2c,d). The photo‐current and photovoltage produced after illuminating the films with 10 and 300 ms light pulses are shown in Figures 2c,d. In both cases, we measured a photovoltage of >0.3 V and photocurrent density of >0.6 mA cm^−2^ when the photodiodes were illuminated at a relatively low light intensity of 40 mW cm^−2^. These values are significantly larger than those measured by single PDCBT or ITIC films (Figure S1, Supporting Information), indicative of the efficient exciton splitting at the PDCBT‐ITIC interface and the formation of free electron carriers. The latter is verified by incorporating these *p*–*n* junctions in a solid‐state organic solar cell (Figure S2, Supporting Information). The large open circuit voltage value obtained (i.e. *V*_OC_=0.9 V) suggests efficient charge photo‐generation and transport processes arising from the optimum optoelectronic properties (e.g., charge mobility, optical absorption) and the highly compatible energetics of the materials used.^[^ ^32^ ^]^ It is reasonable to expect that illuminated PDCBT‐ITIC junctions can generate appreciable amounts of photocurrent and photovoltage in aqueous environments, superior to single film‐based devices. It is also worth noting that the PDCBT‐ITIC junctions show good photo‐response when illuminated with narrow wavelength LED sources (i.e., 470, 530, 655 nm), as shown in Figure S3 (Supporting Information). The wide optical absorption spectra of these junctions in all the visible spectrum (shown in Figure 1) allow for efficient photoexcitation in different wavelengths—a useful feature for wireless optical stimulation biotechnologies.

Further information for the mechanism of the photo‐charge generation by the *p*–*n* heterojunction photodiode operated in PBS can be extracted from photocurrent and photovoltage versus time graphs in Figure 2c,d. First, the polarity of the photocurrent is cathodic; that is, electrons migrate to the semiconductor/electrolyte interface. We observe that both the voltage and the current rapidly increase and reach maximum values in the first few ms after illumination. This fast charging component can be ascribed to the photo‐capacitive effect^[^ ^25^ ^]^—free electrons are generated by the PDCBT‐ITIC junctions and transported rapidly at the ITIC/electrolyte interface and interact electrostatically with cations of the electrolyte. The free electrons cause a capacitive displacement current between the ITIC/electrolyte and the Ag/AgCl/electrolyte interfaces. However, high photocurrent and photovoltage values are sustained for the whole duration of the light illumination, indicative of a continuous faradaic reaction (i.e., oxygen reduction reactions). Photocathodic current is maintained for over 90 min (Figure S4, Supporting Information). Considering previous reports about such photocathodic behavior of organic semiconductor films,^[^ ^35^, ^36^, ^37^ ^]^ we suspect oxygen reduction reactions as the cause for this long‐lasting and high photocurrent of our device. These studies indicate hydrogen peroxide (H_2_O_2_) as the dominant product of photocathodic oxygen reduction (faradaic yield of 90+%).^[^ ^35^, ^36^ ^]^ To verify the validity of this observation for our system, we probed H_2_O_2_ at the *p*–*n* junction upon illumination (Figure S4, Supporting Information) and found a faradaic yield of 159% for photocurrent‐to‐peroxide conversion. Values above 100% are possible only if H_2_O_2_ is produced via an additional pathway besides photocathodic charge transfer. An alternative pathway characterized in organic thin films is photochemical, where photogenerated excitons result in oxygen reduction with concurrent oxidation of some donor, often the organic photoactive material itself.^[^ ^38^ ^]^ To confirm that photochemical H_2_O_2_ evolution indeed happens, we irradiated a film of the *p*–*n* junction on glass (no bias or current source) and found that 27 µm of H_2_O_2_ has been produced, which was measured using a calibrated amperometric probe. These results indicate that photo‐irradiation of PDCBT‐ITIC junctions, lead to H_2_O_2_ generation via a charge transfer of photo‐generated electrons and oxygen, and that an underlying electrical contact is not needed to support peroxide production.^[^ ^35^ ^]^

The proposed mechanisms of operation are illustrated in Figure 2g. Photons form excitons in both PDCBT and ITIC, which are split at the *p*–*n* interface due to their appropiate highest occupied molecular orbital (HOMO) and lowest unoccupied molecular orbital (LUMO) energies, which render the exciton splitting at this interface very efficient. (See Figure S5, Supporting Information, for the ultra‐violet photoelectron spectroscopy (UPS) results for HOMO and LUMO estimation). These electrons are then transported to the ITIC/electrolyte interface and captured by molecular oxygen in the electrolyte to produce H_2_O_2_. When pristine PDCBT or ITIC films are used, excitons recombine to re‐establish the low‐energy ground state,^[^ ^39^ ^]^ leading to significantly lower photocurrent and photovoltage values and low amounts of H_2_O_2_ generated upon illumination (Figure S1, Supporting Information). Overall, the proposed PDCBT‐ITIC junctions are highly photosensitive and can generate H_2_O_2_ via two mechanisms: 1) in photocathodic operation with an underlying electrode and electrochemical circuit, 2) in photochemical operation where neat films on the insulating substrate photochemically reduce oxygen to H_2_O_2_.

Next, we spin‐coated our photo‐sensitive PDCBT and ITIC films on glass coverslips and cultured different types of cells on their surface. An essential step of cell culture is the sterilization of the substrates to avoid cell contamination. Before cell seeding, the polymer films were sterilized by immersing them in 70% ethanol for over 1 h. As shown in Figure S6 (Supporting Information), the ethanol sterilization process did not affect the absorption spectra of the organic semiconducting films, suggesting that their optoelectronic properties were maintained. To evaluate the biocompatibility of the films, we first studied the adhesion and growth of Madin–Darby Canine Kidney cells (MDCK II) on the films. As shown in Figure S7 (Supporting Information), cells cultured directly on the sterilized organic surfaces showed limited spreading on the surface, and cell clusters were formed. This behavior is due to the hydrophobic nature of these films, as verified by the water contact angle measurements shown in Figure S6 (Supporting Information). To enhance cell adhesion and spreading, we coated the thin‐film surfaces with a thin layer of an extracellular matrix protein, i.e., poly‐D‐Lysine (P‐*d*‐L). This adhesion layer reduced the water contact angle from 110^o^ to 62^o^ and from 109^o^ to 58° for PDCBT and ITIC, respectively (Figure S6, Supporting Information), and allowed to form a healthy MDCK II tissue on both organic surfaces. We used the same approach to grow human embryonic kidney cells (HEK 293) on top of PDCBT‐ITIC films, and the healthy state of cells was evidenced by the live/dead assay analysis (Figure S8, Supporting Information). It is also important to note that the modification of the organic surface with P‐*d*‐L does not affect the ability of the materials to produce photovoltage when illuminated with low light intensity, as shown in Figure S9 (Supporting Information). These results prove the biocompatibility of PDCBT and ITIC films and show their potential to be used as platforms for interfacing different cell types while maintaining their optoelectronic properties.

**Figure 3:**
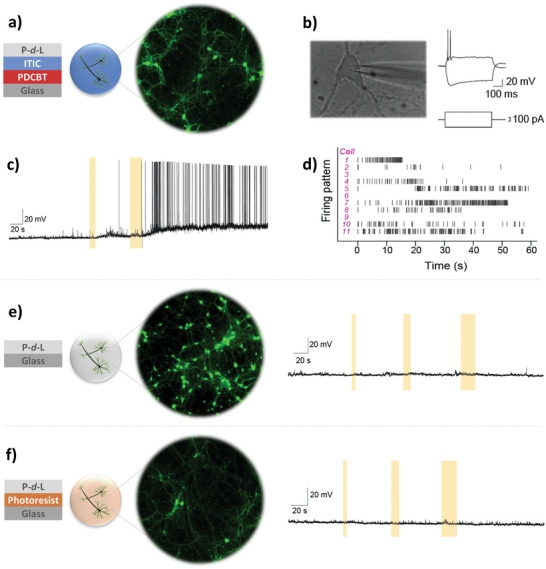
a) Live/Dead assay of mouse cortical neurons stained with calcein AM (green, live cells) and ethidium homodimer‐1 (red, dead cells) cultured on glass coverslips coated with polymer *p*–*n* junctions and P‐*d*‐L. b) a picture of a neuron grown on a PDCBT‐ITIC coated glass coverslip during patch‐clamp measurements and the corresponding voltage time‐course trace, with injection of depolarization and hyperpolarization currents. c) Membrane potential recordings and action potential generation of neurons after several seconds of light exposure (yellow highlighted area). d) Raster plot, showing the action potential (black lines) generated by eleven individual neurons grown on polymer coated glass cover slips, right after light illumination. e) Live/Dead assays of the neurons grown on a glass/P‐*d*‐L substrate, and a representative membrane potential recording of such a neuron exposed to light illumination therein (*n* = 8). f) Live/Dead assays of the neurons grown on a glass cover slip coated with photoresist/P‐*d*‐L substrate, and a representative membrane potential recording of such a neuron exposed to light illumination (*n* = 3).

We then grew cortical neurons extracted from mice, directly on top of glass cover slips coated with PDCBT‐ITIC and P‐*d*‐L. As proven by Live/Dead assays (**Figure** **3**a), healthy primary neuronal networks developed after 14 days in culture, proving the excellent compatibility of these interfaces. To further evaluate the functionality of neurons, we used the well‐established patch clamp technique in current clamp mode.^[^ ^40^ ^]^ The representative voltage time‐course trace of a cortical neuron, grown on the PDCBT‐ITIC film‐coated coverslip (Figure 3b), confirm that the neurons were functional and capable of producing action potentials upon depolarization current injection.

Having ensured the healthy growth of the mouse cortical neurons on the organic junction surface, we utilized the photo‐sensitive properties of PDCBT‐ITIC films to test the possibility of photo‐stimulating the cells. Note that the organic films were directly spin‐coated on the glass coverslips (no ITO or any other underlying conducting substrate); therefore, the experiment was designed to test only for photochemical stimulation effects. We used white light pulses ranging between 5–20 s, at low intensity (40 mW cm^−2^), and, at the same time, monitored the changes in the membrane potential of individual neurons grown on top of PDCBT‐ITIC coated cover slips. As shown in Figure 3c, we observed drastic changes in individual neuron electrophysiology upon light exposure. The membrane potential undergoes a slow and progressive depolarization as a response to light illumination, followed by a burst train of action potential spikes. This measurement clearly indicates that light interferes with neuron‐active electrophysiology via a reaction mediated by PDCBT‐ITIC photosensitive layers. Similar results were obtained from several individual neurons recordings, extracted from >10 different mice, and cultured in several separate experimental batches on PDCBT‐ITIC‐P‐*d*‐L coated cover slips. Upon light exposure, neurons maintained a high firing frequency for several seconds. The reproducibility of this stimulation is shown in Figure 3d (Raster plot). We observed that eight out of eleven individual neurons (i.e., 72%), studied on PDCBT‐ITIC coated cover slips right after light exposure, produce frequent action potential spikes as they are stimulated. Further analysis of these individual neuron signals shows that the average action potential frequency of these neurons ranging from 5 to 60 Hz with a median value of ≈10 Hz (Figure S10, Supporting Information)—the normal firing rate of healthy cortical neurons.^[^ ^41^, ^42^ ^]^ These results suggest that these neurons are functional, also verified by voltage time‐course traces obtained from neurons after been exposed to light irradiation (a representative trace is shown in Figure S11, Supporting Information). We also observed individual neurons (2 out of 11 neurons studied—18%) that showed a similar drastic increase in firing rate, yet with much smaller initial depolarization of the membrane potential (Figure S12, Supporting Information).

While the effect of light on neural activity is clear, we further investigated the stimulation mechanism with a several controlled experiments. First, we performed an identical light stimulation experiment with healthy neurons grown on pristine glass‐ P‐*d*‐L coverslips (Figure 3e). Patch clamp measurements show that these neurons do not respond to light stimulation even after several consecutive, 5–20 s long, light pulses (*n =* 8). We, therefore, eliminate the possibility of the direct interaction of light with neurons without the mediation by the PDCBT‐ITIC films. These control experiments also indicate that light illumination does not create any electrical stimulation artifact in electrophysiology data. We next aimed to understand whether heating, induced by light, could trigger the observed neural function by growing healthy neurons on a glass coverslip coated with a thin layer of photoresist (Figure 3f)—an insulating layer that absorbs light in similar wavelengths as the PDCBT‐ITIC junction and effectively converts light into heat. No membrane potential change and action potential generation were recorded for individual neurons grown on this film under the same illumination conditions (5–20 s of white light at 40 mW cm^−2^, *n* = 3) (Figure 3f). As a result, we suggest that action potentials triggered on neurons cultured on the PDCBT‐ITIC films does not involve a photo‐thermal mechanism.

**Figure 4:**
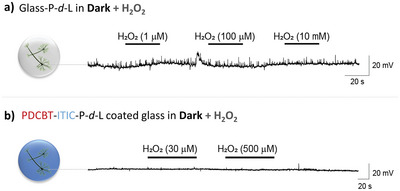
Membrane potential recordings of mouse cortical neurons exposed to different H_2_O_2_ concentrations in the dark. The cells were cultured on a) glass‐ P‐*d*‐L coverslips (*n* = 5) or b) glass ‐PDCBT‐ITIC‐P‐*d*‐L coverslips (*n* = 5).

All the experimental evidence summarized in Figure 3 suggests that photo‐capacitive and photo‐thermal effects cannot be held responsible for the photo‐stimulation of neurons, leaving a photochemical pathway mediated by the polymer films as a plausible mechanism. H_2_O_2_ is a possible chemical that may lead to the electrophysiological response as it is produced by PDCBT‐ITIC junctions upon light exposure, as studied earlier in Figure 2 and Figure S4 (Supporting Information). H_2_O_2_ was, in fact, reported to interact with cell membrane proteins such as TRPV‐1 and TRPV‐4 ion channels.^[^ ^40^ ^]^ In addition, it was found that these channels could be triggered in HEK 293 cells^[^ ^43^ ^]^ as well as epithelial progenitor cells^[^ ^16^ ^]^ by light stimulation mediated by organic semiconductors. We studied the effect of H_2_O_2_ perfused in the neural culture on the films affected their functionality. As shown in **Figure** **4**, the addition of pure H_2_O_2_ (1 µm–10 mm) has no effect on the activity of neurons, grown on both glass‐P‐*d*‐L, and PDCBT‐ITIC‐P‐*d*‐L‐coated glass. Increasing the H_2_O_2_ concentration from 1 µm to 10 mm had no effect on action potential generation, while neurons were no longer functional after prolonged exposure to H_2_O_2_ as shown by the representative voltage‐time course reported in Figure S13 (Supporting Information).

Although we have ruled out direct H_2_O_2_‐based stimulation, other reactive oxygen species or photo‐chemical reactions in the cell media mediated by PDCBT‐ITIC photosensitive junction may cause cell stimulation. We tested whether light or H_2_O_2_ induces any change in organic semiconductor films, which could then lead to the observed membrane potential changes by using NMR (Figures S15 and S16, Supporting Information). ITIC and PDCBT were both covered with cell media for >10 days and subjected to 40 mW cm^−2^ white light irradiation as well as incubated with 500 µm of H_2_O_2_. All H‐NMR peaks remained unaltered in both conditions, indicating excellent stability of the films under light and H_2_O_2_. Therefore, the possibility of a direct chemical release from the organic film network is excluded.

**Figure 5:**
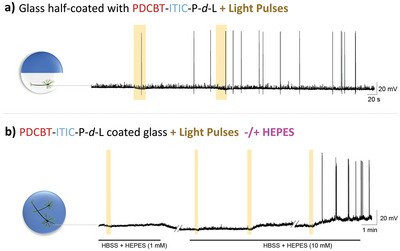
a) Representative membrane potential traces of a mouse cortical neuron cultured on a glass coverslip half‐coated with PDCBT‐ITIC‐P‐*d*‐L. Primary neurons were seeded, maintained, and studied only on the bare glass‐P‐*d*‐L side. b) The traces were recorded from a culture cultured on top of glass ‐PDCBT‐ITIC‐P‐*d*‐L coverslip before and after the addition of HEPES to the culture. The highlighted areas represent the light pulses.

To understand whether a neuron needs to be in direct contact with the semiconducting films for effective light stimulation. We designed “half‐coated” glass coverslips—only a part of the glass coverslip was coated with PDCBT‐ITIC‐P‐*d*‐L, as shown in **Figure** **5**. We cultured primary cortical neurons only on the glass side of this coverslip and performed patch‐clamp measurements during light exposure (Figure 5a). Several individual neurons (*n* = 5) studied on different coverslips showed action potential generation shortly after light exposure. These cells preserved their functionality long after light exposure, as proved by voltage‐time course traces (Figure S14, Supporting Information). These results strongly indicate that primary neurons do not necessarily have to be in direct contact with the organic film to be stimulated by light.

All the above results exclude several possible mechanisms for how light can stimulate neuron electrophysiology and suggest that this can be only induced via PDCBT‐ITIC photochemical reactions with compounds present in the cell culture environment. Our literature search has led us to interesting studies that could potentially be linked to our findings. We found that “Good's buffers”—buffer agents that are used to maintain physiological pH conditions in cell culture media—can be oxidized in the presence of light and several different catalysts, including H_2_O_2_ as well as other oxygen reduction reaction (ORR) species.^[^ ^44^ ^]^ Our cell culture media contains HEPES [4‐(2‐Hydroxyethyl) piperazine‐1‐ethane‐sulfonic acid)], one of the commonly used Good's buffers that is also known to be photo‐oxidized under certain conditions. We performed patch‐clamp measurements on neurons grown PDCBT‐ITIC‐P‐*d*‐L coated glass coverslips and perfused with cell culture media with low HEPES concentration (1 mm—necessary to keep neurons relatively active) and with normal HEPES concentration (10 mm). Low‐intensity white light irradiation was periodically applied under both conditions, and the membrane potential changes were recorded, as shown in Figure 5b. We found that neurons in cell media with low concentration of HEPES did not respond to light pulses—no action potential generation, or membrane potential changes associated with action potentials. When a normal cell culture media, containing 10 mM HEPES, was perfused, the same cell responded to light exposure—the membrane potential depolarized, and action potentials were generated. The corresponding voltage‐course traces are shown in Figure S17 (Supporting Information), confirming the neuron excitability at each stage, and proving the neuron functionality after light exposure. We performed the same type of HEPES experiments with “half coated” glass coverslips as introduced above and observed that the neurons responded similarly (Figure S18, Supporting Information). These results indicate the role of HEPES in the cell culture media as a mediator for neuron photochemical stimulation. We suggest that light causes a direct electron exchange between the photogenerated electrons from PDCBT‐ITIC, to either molecular O_2_ and/or HEPES. We can think about two possible mechanisms of neuron photochemical stimulation: 1) HEPES radicals, which are formed in the presence of H_2_O_2_ and other oxygen reduction reaction (ORR) species (e.g., oxygen superoxide O_2_^−^, Hydroxyl free radicals ^•^OH, peroxynitride ONOO^−^),^[^ ^45^ ^]^ and interact with neuronal proteins to trigger membrane depolarization and action potential generation. These radicals are found to be relatively stable, with half times ranging from 5–30 min in aqueous environments,^[^ ^46^ ^]^ and therefore validate the scenario of chemical delivery of these radicals to neurons that are not in direct contact with the organic semiconductor surface (Figure 5b). Similar response is also reported for neurons exposed to chemical compounds/drugs in vitro, such as, carbachol^[^ ^47^ ^]^ and acetylcholine.^[^ ^48^ ^]^ 2) The reaction of HEPES destabilizes the solution pH and leads to change in the intracellular/extracellular H^+^ signaling balance. Similar neuronal electrophysiology response has also being reported, in response to pH changes with HEPES used as the pH buffering agent.^[^ ^49^ ^]^

## 3 Conclusion

Solution‐processed organic semiconducting heterojunctions made of the p‐type polymer PDCBT and the n‐type small molecule ITIC (a non‐fullerene acceptor) are proposed as highly sensitive photostimulation platforms of primary neurons in vitro. We found that these films convert low‐intensity light into cues through photo‐chemical processes without the need of external electrodes or conducting substrates. We first show the potential of these emerging class of materials to be used in established photo‐stimulation technologies (e.g., photo‐capacitive stimulation) by a detailed investigation of their photo‐electrochemical operation. Bilayer *p*–*n* junctions are preserved when illuminated in aqueous electrolytes, as revealed by photo EQCM‐D measurements in operando and generate a photo‐voltage of >0.3 V and a photo‐current >0.6 mA cm^−2^ when deposited on conducting substrates. Analysis of the photocurrent and photovoltage waveforms combined with measurements of H_2_O_2_ produced, suggest a mixed photo‐capacitive and photo‐electrochemical mechanism – i.e., electron transfer between the semiconductors and molecular O_2_. These results suggest that further optimization of these junctions and integration in microfabricated circuits could provide highly precise photo‐stimulation tools. We then focused on neat organic semiconductor junctions and their photo‐chemical reactions that occur at the semiconductor/cell interface. We found that these drastically affect the electrophysiological activity of primary neurons. Although we found that oxygen reduction reactions and H_2_O_2_ generation are favorable when PDCBT‐ITIC is illuminated, we observed that a direct H_2_O_2_ interaction with primary neurons is not responsible for the changes in neurons’ activity. In addition, we found that neurons do not necessarily need to be in direct contact with the semiconducting films to be affected by light illumination. Our results with “half‐coated” glass slides suggest that photo‐induced chemical gradients are formed in the cell culture media, mediated by photogenerated charges donated by PDCBT‐ITIC junctions. These gradients are most possibly responsible for triggering action potential firing on the primary cortical neurons studied. The role HEPES, is also revealed and suggest that buffer agents, commonly used in in vitro studies might act as mediators for photo‐stimulation via reactions with reactive oxygen species and photo‐generated electrons by photosensitive organic semiconducting films.

Although the exact photo‐chemical reactions cannot be revealed due to the advanced complexity of the system, the proposed stimulation pathways presented in this study offer new insights in the field of light‐sensitive platforms for wireless control of neuronal activity. The possibility of photo‐generated species created with direct light/organic semiconductor interactions, that can interfere with cell activity create new opportunities for light‐based therapies mediated by organic semiconductors.

## 4 Experimental Section

### Materials

Glass‐ITO substrates (sheet resistance 15 Ω sq^−1^) were purchased from Xin Yan Technology LTD. PDCBT was purchased from 1‐material inc. ITIC was purchased by Ossila ltd. o‐IDTBR was synthesized following the synthetic procedure reported by Holiday et al.^[^ ^50^ ^]^

### Device Fabrication

Glass substrates fully coated with indium tin oxide (ITO) were cleaned by sonication in acetone and isopropanol, followed by oxygen plasma treatment. The p‐type PDCBT layer was deposited from 10 mg ml^−1^ solution in chlorobenzene by spin coating at 2000 rpm resulting in a thickness of 40 nm. The solution was heated at 80 °C before spin coating and spin‐coated hot to ensure good layer homogeneity. ITIC was spin‐coated on top of PDCBT layers at 2000 rpm from 10 mg ml^−1^ solutions in toluene, resulting in a thickness of 40 nm. For neat PDCBT‐ITIC junctions we followed the same protocol to coat the films on round glass cover slips (D = 12 mm). For “half‐coated” glass cover slips, half of the surface of the glass slide was gently removed by wiping the polymers off with acetone. Organic solar cell devices were fabricated with a *p*–*n* device architecture (NiO/PDCBT/ITIC). NiO was prepared using a sol‐gel method on ITO substrates reported elsewhere, followed by the sequential deposition of the *p*–*n* layers. Finally, a 100 nm thick aluminum layer was thermally evaporated on top of the active layers of the devices through a shadow mask yielding active areas of 0.045 cm^2^ on each device.

### Ultraviolet Photoelectron Spectroscopy (UPS) and Energy Bands Calculation

UPS measurements were recorded using a SPECS PHOIBOS 100 hemispherical electron energy analyzer in a custom‐built ultrahigh vacuum system with a base pressure of ≈1 × 10^−10^ mbar. Samples were excited at 21.21 eV using a He I plasma source. UPS measurements were performed on polymer films spin‐coated on a gold foil identically to the ones used for the photo‐stimulation experiments. The work function (WF) for each film was calculated by subtracting the He I radiation energy of 21.2 eV from the high‐binding‐ energy cutoff at 16.4 eV for PDCBT and 17 eV for ITIC (Figure S4, Supporting Information). Therefore, the WF of PDCBT was found at 21.2 – 16.4 = 4.8 eV and the WF of ITIC was found at 21.2 – 17 = 4.2 eV versus vacuum. The valence band maximum (LUMO) was found at 3.3 eV for PDCBT and 3.9 eV for ITIC (Figure S4, Supporting Information). The HOMO value was estimated at 5.2 eV for PDCBT and 5.6 eV for ITIC, after subtracting the energy band gap (calculated from optical absorption spectra) from the LUMO values for each film.

### Photochemical Characterization and Measuring H_2_O_2_ Concentrations

Measurements of photovoltage and photocurrent of the *p*–*n* junction‐based devices were conducted inside a dark Faraday cage, and the electrophotoresponse (EPR) data were collected via a high‐resolution 15‐bit two‐channel PicoScope 5243B oscilloscope as described previously.^[^ ^30^ ^]^ Briefly, the backside ITO of the OEPC was contacted with a probe electrode connected to the positive terminal of an oscilloscope. Meanwhile, the negative terminal was connected to an Ag/AgCl electrode in 0.1 M KCl electrolyte, making contact with the top of the organic layer. The intensity of the LED used (wavelength = 630 nm) is 0.33 mW mm^−2^. Local H_2_O_2_ evolution was measured in situ using a 4‐channel micro‐amperometric amplifier system (TBR4100, World Scientific Instruments) with a 4‐channel ADC board (LabTrax, World Scientific Instruments). The respective sensor probe used was ISO‐HPO‐2. The sensor was constantly polarized at a bias of 450 mV and calibrated before the measurement following the procedure reported in the instruction manual. The increase in H_2_O_2_ concentration was recorded using LabScribe software (World Scientific Instruments). The experiment was carried out by placing the sample in a PBS‐containing vial and irradiating it with a white light LED of 942 mW optical power for 3 h. Next, a micro‐amperometric sensor (Clark electrode) has been placed in the vial to detect the amount of H_2_O_2_ evolved after 3 h of reaction. The concentration of H_2_O_2_ evolved after the reaction was 27 µM. Longer measurements of photocathodic behavior shown in Figure S3 (Supporting Information) were conducted using an Ivium Vertex One potentiostat and a three‐electrode sealed photo‐electrochemical cell, with peroxide concentration quantified using a photometric assay with tetramethylbenzidine as described in Ref.[51] To measure the thickness of the conjugated polymer thin films, a Dektak‐Veeco stylus profilometer was used. Current density versus voltage (*J–V*) characteristics of the solar cells under study was measured by using a Xenon lamp at AM1.5 solar illumination (Oriel Instruments) calibrated to a silicon reference cell with a Keithley 2400 source meter, and a custom‐made LabVIEW software. Optical absorption profiles were recorded with an Ocean Optics QE65 Pro Spectrometer, Ocean Optics HL‐2000‐FHSA halogen light source, OceanView software, and a sample holder purchased from redox.me (MM Spectro‐EFC). Water contact angle measurements were performed using the sessile drop method with a KRUSS instrument.

### Electrochemical Quartz Crystal Microbalance with Dissipation Monitoring

We performed EQCM‐D measurements using a Q‐sense analyzer (QE401, Biolin Scientific). Thickness and swelling measurements were performed as follows. First, we recorded the QCM‐D response of the bare Au sensors in the air, followed by the injection of the PBS 1X solutions into the chamber. This resulted in large shifts in frequency (*f*) and dissipation of energy (*D*), due to the density differences between the two media. The measurements were then stopped, the sensors were removed, and the PCDBT polymer film was spun cast directly on the same sensor from a 10 mg/mL hot chlorobenzene solution at 2000 rpm. The absolute *f* value for the polymer‐coated sensor was obtained in air, and PBS 1x after the f signal was perfectly flat (i.e., f <0.5 Hz), assuring that the system is in equilibrium. The sensor was removed again, ITIC film was spun cast from a 10 mg/mL toluene solution at 2000 rpm, and inserted back in the chamber to measure the absolute f in air and PBS 1X. We then compared the absolute difference in *f* for multiple overtones between the bare sensor and the polymer‐coated sensor, both in air and in PBS 1X, using the function “stitched data” of Q‐soft software. This function compares the selected datasets based on the raw frequencies measured and excludes the effect of the different densities between the two media. Thus, the difference in the *f* values of the stitched data is directly analogous to the thickness of the polymer in both media, which is calculated by using the Sauerbrey equation: $\Delta m\;=\;\frac{-17.7}{n}{\;\Delta f}_{n}$.

Q‐Tools and D‐find software were used for the modeling and data analysis. After the swelling measurements, an Autolab PGstat128N potentiostat was coupled with a Q‐sense electrochemistry module, and the current was recorded. The three‐electrode setup comprised Ag/AgCl reference, Pt counter, and Au/polymer EQCM‐D sensor as the working electrode. An optical fiber was attached to the window of the module, and white light pulses were applied with an Ocean Optics HL‐2000‐FHSA halogen light source. The current was simultaneously recorded with the potentiostat, while the raw frequency changes were recorded with the QCM‐D. The changes in the thickness (or mass) of the polymer films and the photocurrents generated could, thus, be extracted *in‐operando*.

### Cell Culture

Human Embryonic Kidney cells (HEK 293) and Madin‐Darby Canine Kidney cells (MDCK II) were cultured in DMEM supplemented with 10% fetal bovine serum, 2 mM Glutamax and 0.5% PenStrep 100X (10 000 U.mL^−1^ Penicillin, 10 000 µg.mL^−1^ Streptomycin). All reagents were purchased from Invitrogen. Cells were routinely maintained in incubators at 37°C and 5% CO_2_. All substrates used for cell culture were sterilized using 70% ethanol for 30 min and rinsed with water. After sterilization, a 500 µg/ml Poly‐d‐Lysine solution in DI water was dropped on the surface of the substrates and left overnight at 4 °C. Prior seeding the substrates were transferred in a sterilized flow hood and rinsed with PBS. Primary cortical neurons were collected from mice. They were handled in accordance with the animal research advisory committee guidelines of the National Institutes of Health (NIH), and procedures were approved by the Institutional Animal Care and Use Committee (IACUC) of King Abdullah University of Science and Technology (KAUST). Primary culture of cortical neurons was prepared from embryonic day 17 CD‐1 IGS mouse embryos (Charles River, UK). Cerebral cortical tissue was dissected, collected and incubated 30 min at 37°C in Hank's Balanced Salt Solution (HBSS) supplemented with papain (20 units.mL^−1^; Worthington Biochemical), L‐7 cysteine (1 mM; Sigma) and DNase I (5 mg.mL^−1^; Worthington Biochemical). Cortical cells were mechanically dissociated and filtered using a 40 µm cell strainer (Fischerbrand) and centrifuged for 5 min at 200xg. The supernatant was carefully removed, and cells were re‐suspended in Neurobasal medium (Gibco) completed with B27 supplement (Gibco), 500 µM L‐glutamine (Sigma), and 0.5x penicillin/streptomycin solution (Gibco). 20 µL of media containing 10^5^ cells were seeded on the device and incubated between 15 and 30 min until neurons settled. Then, 1 mL of warm (37°C) cell media was carefully added to the well. After 4 h, the media was replaced to remove dead cells. Neurons were incubated at 37°C, 95% humidity, and 5% CO_2_. Media were changed twice a week and replaced with half the volume of a fresh batch.

### Live/Dead Assay

Cells were seeded on top of the device and incubated for 24–48 h for epithelial cells and 14 days for cortical neurons. The media was removed and a mixture of calcein‐AM, propidium iodide (Sigma‐Aldrich) was applied. After 5–15 min of incubation, devices were rinsed three times with HBSS (Invitrogen), and cells were monitored using an inverted fluorescent microscope (DMi8, Leica Microsystems).

### Whole cell Patch Clamp Experiments

Primary cortical neurons were visualized using an inverted microscope Carl Zeiss Axio Observer.A1 (Carl Zeiss, Oberkochen, Germany). Neurons with classical pyramidal shape were chosen to perform the experiments. The experiments were done under minimal light conditions in a dark room to prevent the activation of the photosensitive devices before patch‐clamping the cells. Patch pipettes were pulled from borosilicate capillaries using a P‐1000 Flaming Brown puller (Sutter Instruments) to a final resistance of 4–5 MΩ when filled with a solution with the following composition (in mm): K^+^‐Gluconate 135, KCl 10, EGTA 1, CaCl_2_ 0.1, HEPES 10, Mg‐ATP 5, Na_2_‐GTP 0.5; pH = 7.2‐7.3 adjusted with HCl. Current clamp recordings were done at room temperature and obtained with an Axopatch 700B amplifier (Axon Instruments, Molecular Devices, Sunnyvale, CA) and digitized at 20 kHz and low pass filtered at 10 kHz (Digidata 1550, Molecular Devices. Most experiments were in normal HEPES‐based solution with the following composition (in mM): NaCl 135, KCl 2.5, CaCl_2_ 2, MgCl_2_ 1, HEPES 10, glucose 10; final osmolarity ≈300 mOsm L^−1^ adjusted with sucrose.). Once the gigaseal was broken and the whole cell configuration was achieved, we recorded the resting membrane potential (RMP) and ran an I/V curve by injecting square current pulses (from −200 in 50 pA steps, 1 sec) until eliciting at least one action potential. Cells with RMP more depolarized than ‐50 mV were discarded from this study. A gap‐free protocol was continuously recorded, and after a stable baseline, the light (intensity 40 mW cm^−2^) was turned on for 1, 2, 5, 10, and 20 s to directly illuminate the patched neuron. All data were acquired and analyzed offline with the pClamp 10.7 software (Molecular Devices). To study the mechanism underlying the light induced increase in neuronal excitability, we ran the following experiments: 1) H_2_O_2_ treatment: We perfused H_2_O_2_ diluted in HBSS at different concentrations ranging 1–10 mm in cells patched on top of glass‐ P‐*d*‐L; of this range we chose 30 and 500 µm to test on cells cultures on PDCBT‐ITIC‐P‐*d*‐L coverslip. The solutions containing H_2_O_2_ were freshly prepared and perfused, after a stable baseline, at a rate of 1.5 ml min^−1^. H_2_O_2_ treatments were performed in the dark. 2) “Half‐coated” glass coverslips: For these experiments, we designed half coated PDCBT‐ITIC‐P‐*d*‐L coverslips, and the neurons were carefully cultured on the top of the glass‐P‐*d*‐L coverslip. Before the patch clamp experiment, we interrupted the neural network between PDCBT‐ITIC‐P‐*d*‐L and glass‐P‐*d*‐L sides to prevent any influence by direct stimulation of dendrites during light exposure. 3) HEPES free saline solution treatment. For the last experiments, we treated the cells cultured on top of PDCBT‐ITIC‐P‐*d*‐L coverslips with a saline solution of the following composition (in mM): NaCl 135, KCl 2.5, CaCl_2_ 2, MgCl_2_ 1, HEPES 1, glucose 10; final osmolarity ≈300 mOsm L^−1^ adjusted with sucrose during light exposure. Then we changed to normal HBSS and exposed to light again.

## Conflict of Interest

The authors declare no conflict of interest.

## Author Contributions

A.S. conceived the research, fabricated the devices, performed EQCM‐D, optical absorption, photo‐electrochemical, and contact angle measurements, assisted with biocompatibility and patch clamp studies, and wrote the manuscript. A.H. performed cell culture, calcium imaging, and live/dead assays with assistance from N.S. and M.K. G.H.L, H.F., and P.M. G.H.L together with A.S. and N.S. performed patch‐clamp measurements and analyzed the results. N.G., D.B., and I.M. selection of materials and assisted with device fabrication. L.M. and E.D.G. performed photoelectrochemical measurements and peroxide quantification. T.S. and R.S. performed patch clamp measurements, analyzed, and interpret the data. A.S., E.D.G., and S.I. designed the experiments and supervised the work. All authors discussed the results and assisted in manuscript input.

## Supporting information

Supporting Information

## Data Availability

The data that support the findings of this study are openly available in biorxiv at https://doi.org/10.1101/2022.02.17.480608, reference number 480608.

## References

[1] M. R. Hamblin , S. T. Nelson , J. R. Strahan , Photobiomodulation, Photomed., Laser Surg. 2018, 36, 241. 29466089 10.1089/pho.2017.4401 PMC5946726

[2] W.‐M. Chan , D. S. C. Lam , T. Y. Y. Lai , D. T. L. Liu , K. K. W. Li , Y. Yao , T.‐H. Wong , Ophthalmology 2004, 111, 1576. 15288991 10.1016/j.ophtha.2003.12.056

[3] J. F. Maya‐Vetencourt , D. Ghezzi , M. R. Antognazza , E. Colombo , M. Mete , P. Feyen , A. Desii , A. Buschiazzo , M. Di Paolo , S. Di Marco , F. Ticconi , L. Emionite , D. Shmal , C. Marini , I. Donelli , G. Freddi , R. Maccarone , S. Bisti , G. Sambuceti , G. Pertile , G. Lanzani , F. Benfenati , Nat. Mater. 2017, 16, 681. 28250420 10.1038/nmat4874 PMC5446789

[4] M. Berggren , E. D. Głowacki , D. T. Simon , E. Stavrinidou , K. Tybrandt , Chem. Rev. 2022, 122, 4826. 35050623 10.1021/acs.chemrev.1c00390 PMC8874920

[5] S. Rohringer , W. Holnthoner , S. Chaudary , P. Slezak , E. Priglinger , M. Strassl , K. Pill , S. Mühleder , H. Redl , P. Dungel , Sci. Rep. 2017, 7, 10700. 28878330 10.1038/s41598-017-11061-y PMC5587748

[6] R. Chen , F. Gore , Q.‐A. Nguyen , C. Ramakrishnan , S. Patel , S. H. Kim , M. Raffiee , Y. S. Kim , B. Hsueh , E. Krook‐Magnusson , I. Soltesz , K. Deisseroth , Nat. Biotechnol. 2021, 39, 161. 33020604 10.1038/s41587-020-0679-9 PMC7878426

[7] D. Maiti , X. Tong , X. Mou , K. Yang , Front Pharmacol 2019, 9, 1401. 30914959 10.3389/fphar.2018.01401 PMC6421398

[8] J. B. Vines , J.‐H. Yoon , N.‐E. Ryu , D.‐J. Lim , H. Park , Front Chem 2019, 7, 167. 31024882 10.3389/fchem.2019.00167 PMC6460051

[9] R. Parameswaran , J. L. Carvalho‐de‐Souza , Y. Jiang , M. J. Burke , J. F. Zimmerman , K. Koehler , A. W. Phillips , J. Yi , E. J. Adams , F. Bezanilla , B. Tian , Nat. Nanotechnol. 2018, 13, 260. 29459654 10.1038/s41565-017-0041-7 PMC6029690

[10] Y. Jiang , X. Li , B. Liu , J. Yi , Y. Fang , F. Shi , X. Gao , E. Sudzilovsky , R. Parameswaran , K. Koehler , V. Nair , J. Yue , K. Guo , Y. Fang , H.‐M. Tsai , G. Freyermuth , R. C. S. Wong , C.‐M. Kao , C.‐T. Chen , A. W. Nicholls , X. Wu , G. M. G. Shepherd , B. Tian , Nat. Biomed. Eng. 2018, 2, 508. 30906646 10.1038/s41551-018-0230-1 PMC6430241

[11] M. Silverå‐Ejneby , M. Jakešová , J. J. Ferrero , L. Migliaccio , Z. Zhao , M. Berggren , D. Khodagholy , V. Đerek , J. Gelinas , E. D. Głowacki , bioRxiv 2020, 2020.07.01.182113.

[12] N. Martino , P. Feyen , M. Porro , C. Bossio , E. Zucchetti , D. Ghezzi , F. Benfenati , G. Lanzani , M. R. Antognazza , Sci. Rep. 2015, 5, 8911. 25753132 10.1038/srep08911 PMC4354102

[13] C. Tortiglione , M. R. Antognazza , A. Tino , C. Bossio , V. Marchesano , A. Bauduin , M. Zangoli , S. V. Morata , G. Lanzani , Sci. Adv. 2017, 3, e1601699. 28138549 10.1126/sciadv.1601699 PMC5266477

[14] D. Ghezzi , M. R. Antognazza , R. MacCarone , S. Bellani , E. Lanzarini , N. Martino , M. Mete , G. Pertile , S. Bisti , G. Lanzani , F. Benfenati , Nat. Photonics 2013, 7, 400. 27158258 10.1038/nphoton.2013.34 PMC4855023

[15] V. Benfenati , N. Martino , M. R. Antognazza , A. Pistone , S. Toffanin , S. Ferroni , G. Lanzani , M. Muccini , Adv. Healthcare Mater. 2014, 3, 306. 10.1002/adhm.201300179 23966220

[16] F. Lodola , V. Rosti , G. Tullii , A. Desii , L. Tapella , P. Catarsi , D. Lim , F. Moccia , M. R. Antognazza , Sci. Adv. 2019, 5, eaav4620. 31598549 10.1126/sciadv.aav4620 PMC6764832

[17] M. Zangoli , F. Di Maria , E. Zucchetti , C. Bossio , M. R. Antognazza , G. Lanzani , R. Mazzaro , F. Corticelli , M. Baroncini , G. Barbarella , Nanoscale 2017, 9, 9202. 28650487 10.1039/c7nr01793f

[18] I. Abdel Aziz , M. Malferrari , F. Roggiani , G. Tullii , S. Rapino , M. R. Antognazza , iScience 2020, 23, 101091. 32438318 10.1016/j.isci.2020.101091 PMC7240120

[19] M. R. Antognazza , I. Abdel Aziz , F. Lodola , Oxid Med Cell Longevity 2019, 2019, 2867516. 10.1155/2019/2867516 PMC6462332 31049131

[20] M. Moros , A. Lewinska , G. Onorato , M. R. Antognazza , M. Di Francesca , M. Blasio , G. Lanzani , A. Tino , M. Wnuk , C. Tortiglione , MRS Commun. 2018, 8, 918.

[21] J. Hopkins , L. Travaglini , A. Lauto , T. Cramer , B. Fraboni , J. Seidel , D. Mawad , Adv. Mater. Technol. 2019, 4, 1800744.

[22] M. Silverå Ejneby , L. Migliaccio , M. Gicevičius , V. Đerek , M. Jakešová , F. Elinder , E. D. Głowacki , Adv. Mater. Technol. 2020, 5, 1900860.

[23] M. Knupfer , Appl. Phys. A 2003, 77, 623.

[24] D. Rand , M. Jakešová , G. Lubin , I. Vėbraitė , M. David‐Pur , V. Đerek , T. Cramer , N. S. Sariciftci , Y. Hanein , E. D. Głowacki , Adv. Mater. 2018, 30, 1707292. 10.1002/adma.201707292 29717514

[25] T. Paltrinieri , L. Bondi , V. Đerek , B. Fraboni , E. D. Głowacki , T. Cramer , Adv. Funct. Mater. 2021, 31, 2010116.

[26] V. Ðerek , D. Rand , L. Migliaccio , Y. Hanein , E. D. Głowacki , Front Bioeng Biotechnol 2020, 8, 284. 32363183 10.3389/fbioe.2020.00284 PMC7180391

[27] S. Vaquero , C. Bossio , S. Bellani , N. Martino , E. Zucchetti , G. Lanzani , M. R. Antognazza , J. Mater. Chem. B 2016, 4, 5272. 32263608 10.1039/c6tb01129b

[28] S. B. Srivastava , R. Melikov , E. Yildiz , U. M. Dikbas , S. Sadeghi , I. H. Kavakli , A. Sahin , S. Nizamoglu , J. Mater. Chem. C 2021, 9, 1755.

[29] T. Schmidt , M. Jakešová , V. Đerek , K. Kornmueller , O. Tiapko , H. Bischof , S. Burgstaller , L. Waldherr , M. Nowakowska , C. Baumgartner , M. Üçal , G. Leitinger , S. Scheruebel , S. Patz , R. Malli , E. D. Głowacki , T. Rienmüller , R. Schindl , Adv. Mater. Technol. 2022, 7, 2101159. 37064760 10.1002/admt.202101159 PMC10097427

[30] M. Jakešová , M. Silverå Ejneby , V. Đerek , T. Schmidt , M. Gryszel , J. Brask , R. Schindl , D. T. Simon , M. Berggren , F. Elinder , E. D. Głowacki , Sci. Adv. 2019, 5, eaav5265. 30972364 10.1126/sciadv.aav5265 PMC6450690

[31] D. Ghezzi , M. R. Antognazza , M. Dal Maschio , E. Lanzarini , F. Benfenati , G. Lanzani , Nat. Commun. 2011, 2, 166. 21266966 10.1038/ncomms1164

[32] A. Wadsworth , M. Moser , A. Marks , M. S. Little , N. Gasparini , C. J. Brabec , D. Baran , I. McCulloch , Chem. Soc. Rev. 2019, 48, 1596. 29697109 10.1039/c7cs00892a

[33] L. Benov , Med Princ Pract 2015, 24, 14. 24820409 10.1159/000362416 PMC6489067

[34] A. Savva , S. Wustoni , S. Inal , J. Mater. Chem. C 2018, 6, 12023.

[35] M. Jakešová , D. H. Apaydin , M. Sytnyk , K. Oppelt , W. Heiss , N. S. Sariciftci , E. D. Głowacki , Adv. Funct. Mater. 2016, 26, 5248.

[36] L. Migliaccio , M. Gryszel , V. Đerek , A. Pezzella , E. D. Głowacki , Mater. Horiz. 2018, 5, 984.

[37] G. M. Suppes , P. J. Fortin , S. Holdcroft , J. Electrochem. Soc. 2015, 162, H551.

[38] M. Gryszel , M. Sytnyk , M. Jakešová , G. Romanazzi , R. Gabrielsson , W. Heiss , E. D. Głowacki , ACS Appl. Mater. Interfaces 2018, 10, 13253. 29624365 10.1021/acsami.8b01295

[39] L. A. Ribeiro , P. H. Oliveira Neto , W. F. da Cunha , L. F. Roncaratti , R. Gargano , D. A. da Silva Filho , G. M. e Silva , J. Chem. Phys. 2011, 135, 224901. 22168721 10.1063/1.3665392

[40] E. Neher , B. Sakmann , Nature 1976, 260, 799. 1083489 10.1038/260799a0

[41] M. Häusser , I. M. Raman , T. Otis , S. L. Smith , A. Nelson , S. du Lac , Y. Loewenstein , S. Mahon , C. Pennartz , I. Cohen , Y. Yarom , J. Neurosci. 2004, 24, 9215. 15496653 10.1523/JNEUROSCI.3375-04.2004 PMC6730100

[42] E. Dégenètais , A.‐M. Thierry , J. Glowinski , Y. Gioanni , Cereb Cortex 2002, 12, 1. 11734528 10.1093/cercor/12.1.1

[43] F. Lodola , N. Martino , G. Tullii , G. Lanzani , M. R. Antognazza , Sci. Rep. 2017, 7, 8477. 28814817 10.1038/s41598-017-08541-6 PMC5559550

[44] G. Zhao , N. D. Chasteen , Anal. Biochem. 2006, 349, 262. 16289439 10.1016/j.ab.2005.10.005

[45] J. K. Grady , N. D. Chasteen , D. C. Harris , Anal. Biochem. 1988, 173, 111. 2847586 10.1016/0003-2697(88)90167-4

[46] M. Kirsch , E. E. Lomonosova , H.‐G. Korth , R. Sustmann , H. de Groot , J. Biol. Chem. 1998, 273, 12716. 9582295 10.1074/jbc.273.21.12716

[47] B. Carette , J Neurophysiol 1998, 80, 1042. 9744920 10.1152/jn.1998.80.3.1042

[48] J. Yamada‐Hanff , B. P. Bean , J. Neurosci. 2013, 33, 15011. 24048831 10.1523/JNEUROSCI.0577-13.2013 PMC3776055

[49] J. W. Deitmer , M. Szatkowski , J Physiol 1990, 421, 617. 2112195 10.1113/jphysiol.1990.sp017965 PMC1190105

[50] S. Holliday , R. S. Ashraf , A. Wadsworth , D. Baran , S. A. Yousaf , C. B. Nielsen , C.‐H. Tan , S. D. Dimitrov , Z. Shang , N. Gasparini , M. Alamoudi , F. Laquai , C. J. Brabec , A. Salleo , J. R. Durrant , I. McCulloch , Nat. Commun. 2016, 7, 11585. 27279376 10.1038/ncomms11585 PMC4906164

[51] M. Gryszel , A. Markov , M. Vagin , E. D. Głowacki , J. Mater. Chem. A 2018, 6, 24709.

