# Supplementary material for: Photo‐Chemical Stimulation of Neurons with Organic Semiconductors
Source: Adv Sci (Weinh). 2023 Sep 3;10(31):2300473. doi: 10.1002/advs.202300473 (PMC10625067; doi:10.1002/advs.202300473)
Supplement: Supplementary file 1 — Supporting Information [file ADVS-10-2300473-s001.pdf]

ADVANCED SCIENCE
Open Access

Supporting Information

for *Adv. Sci.*, DOI 10.1002/advs.202300473

Photo-Chemical Stimulation of Neurons with Organic Semiconductors

Achilleas Savva, Adel Hama, Gabriel Herrera-López, Tony Schmidt, Ludovico Migliaccio, Nadia Steiner, Malak Kawan, Hubert Fiumelli, Pierre J. Magistretti, Iain McCulloch, Derya Baran, Nicola Gasparini, Rainer Schindl, Eric D. Głowacki and Sahika Inal**

Supporting Information

Photo-chemical stimulation of neurons with organic semiconductors

Achilleas Savva,* [1,2] Adel Hama,[1] Gabriel Herrera-López,[1] Tony Schmidt,[3] Ludovico Migliaccio,[4] Nadia Steiner,[1] Malak Kawan,[1] Hubert Fiumelli,[1] Pierre J. Magistretti,[1] Iain McCulloch,[5] Derya Baran,[5] Nicola Gasparini,[6] Rainer Schindl,[3] Eric D. Głowacki,[6] and Sahika Inal* [1]

[1] *Biological and Environmental Science and Engineering, King Abdullah University of Science and Technology (KAUST), Thuwal 23955-6900, Saudi Arabia.*
[2] *Department of Chemical Engineering and Biotechnology, University of Cambridge, CB30AS Cambridge, United Kingdom.*
[3] *Gottfried Schatz Research Center, Chair of Biophysics, Medical University of Graz, Neue Stiftingtalstraße 6, Graz 8010, Austria.*
[4] *Bioelectronics Materials and Devices Laboratory, Central European Institute of Technology, Brno University of Technology, Purkyňova 123, 61200 Brno, Czech Republic.*
[5] *Physical Science and Engineering (PSE), KAUST Solar Center (KSC), King Abdullah University of Science and Technology (KAUST), Thuwal 23955-6900, Saudi Arabia.*
[6] *Department of Chemistry and Centre for Processable Electronics, Imperial College London, W12 0BZ, UK.*

**Corresponding authors: as3024@cam.ac.uk; sahika.inal@kaust.edu.sa*

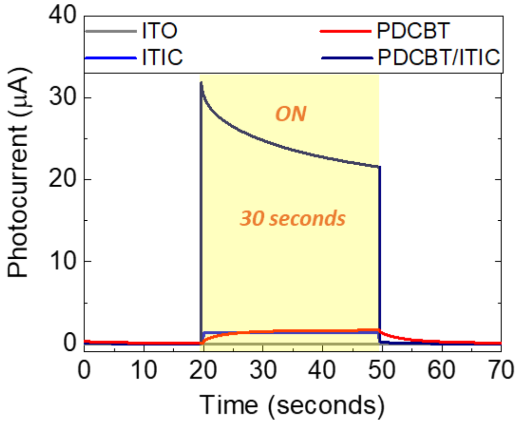

Figure S1: Photocurrent measurements of ITO only (grey line) ITO/PDCBT (red line) ITO/ITIC (blue line) ITO/PDCBT/ITIC (dark blue line) upon illumination with white light at 40 mW/cm^2.

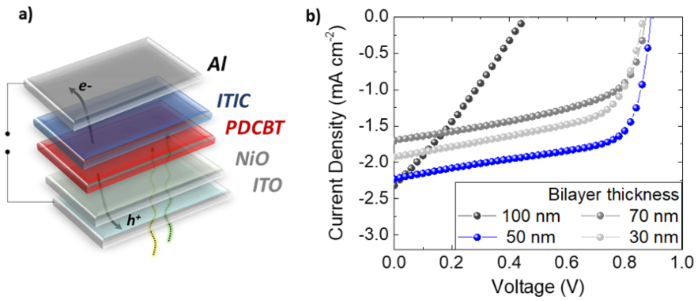

Figure S2: **a)** Schematic of the bilayer organic solar cell devices used - ITO/NiO/PDBCT/ITIC/Al. **b)** Current density versus voltage characteristics of the organic solar cells measured with different overall PDCBT-ITC thickness. The maximum short circuit current (2.2. mA/cm^2) as well as the maximum open-circuit voltage (0.9 V) is achieved with a total PDCBT-ITIC thickness of 50 nm.

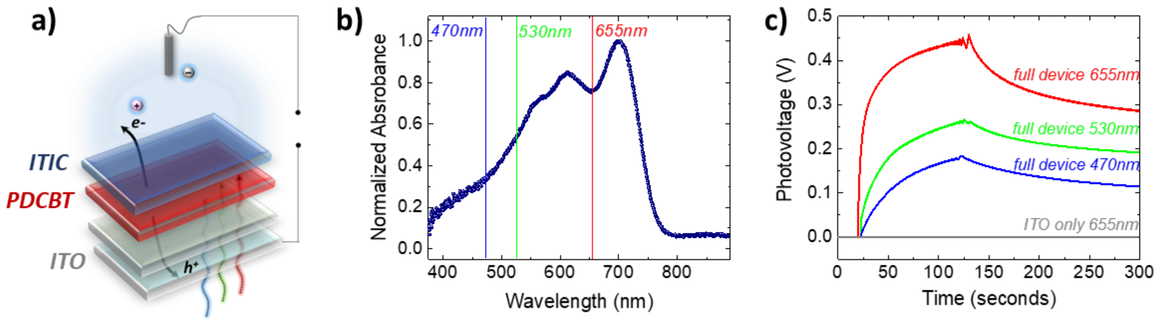

Figure S3: **a)** A schematic of the setup used to study the photo-response of the diodes in PBS 1X. **b)** The optical absorption spectra of the diodes and the wavelengths of LED's used for photo-esxitation and **c)** the photovoltage response of the system when photoexcited at different wavelengths. In all cases the applied light pulse was 100 seconds long with an intensity of 100 mW/cm^2.

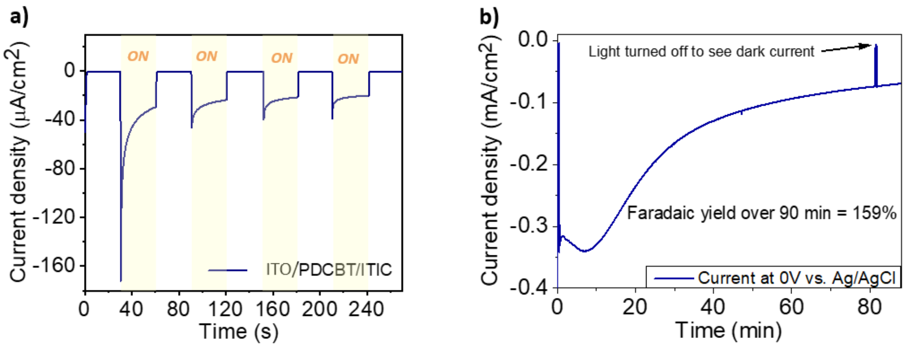

Figure S4: **a)** Photocurrent generated upon 4 consecutive, 30 second-long white light pulses (40 mW/cm^2) **b)** Photo(electro)catalysis of a representative sample ITO/PDCBT/ITIC illuminated with a halogen lamp as light source; E[V] = 0 Vt = 5400s.

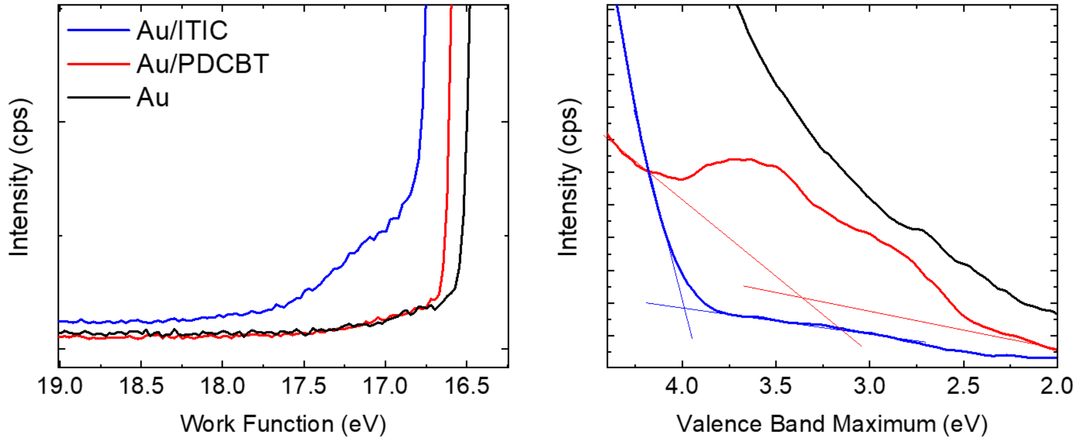

<u>Figure S5</u>: Ultraviolet photoelectron spectroscopy measurements of the polymer semiconductors thin films used in this study, i.e. PDCBT (p-type), ITIC (n-type).

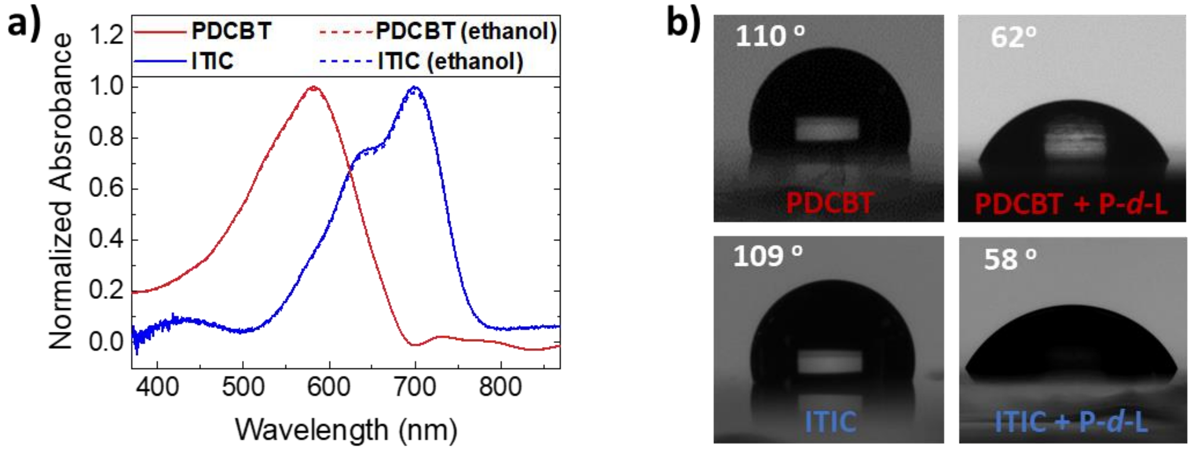

Figure S6: a) Optical absorption of the PDCBT (red) and ITIC (blue) thin films as cast (solid lines) and after sterilization with 70% ethanol for 1 hour (dashed lines) **b)** Water contact angle of the PDCBT and ITIC films before and after coating their surface with poly-d-lysine (P-*d*-L).

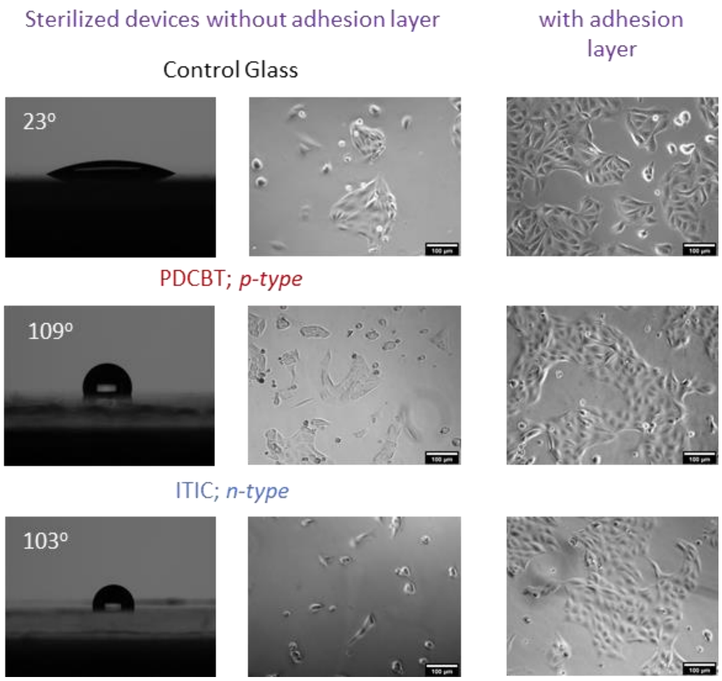

Figure S7: Madin-Darby Canine Kidney cells (MDCK II) cells cultured on the surface of control glass substrates (top raw), on PDCBT surface (middle raw) and on ITIC surface (bottom raw). The use of a thin adhesion layer (rat tail collagen) improves cell adhesion on the polymer surfaces and the formation of an epithelial layer. Scale bars = 100 µm.

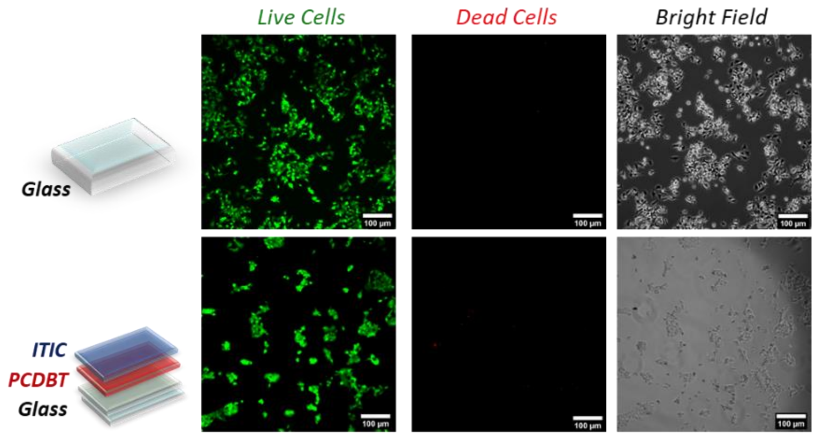

Figure S8: Live/dead assay of Human Embryonic Kidney cells (HEK 293) cells cultured on the surface of control glass substrates (top raw), and on PDCBT/ITIC surface (bottom raw). Scale bars = 100 µm.

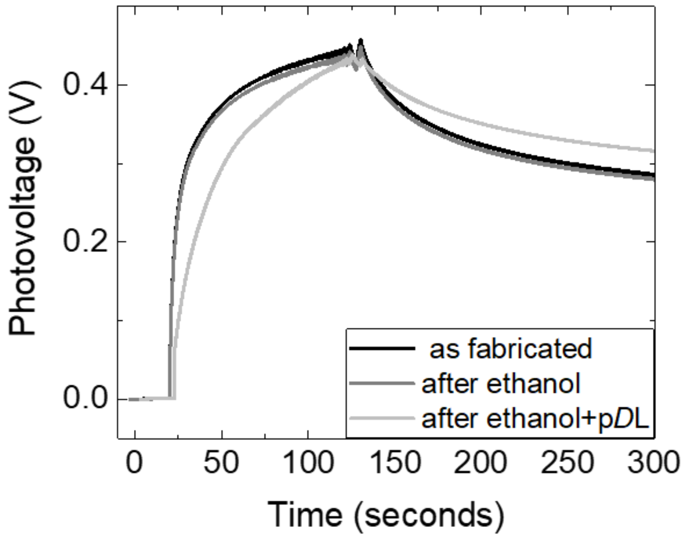

Figure S9: Photovoltage measurements of PDCBT/ITIC right after fabrication (grey line), after ethanol sterilization (dark grey line) and after surface modification with P-*d*-L. All samples were immersed in PBS 1X and illuminated with white light at 40 mW/cm^2.

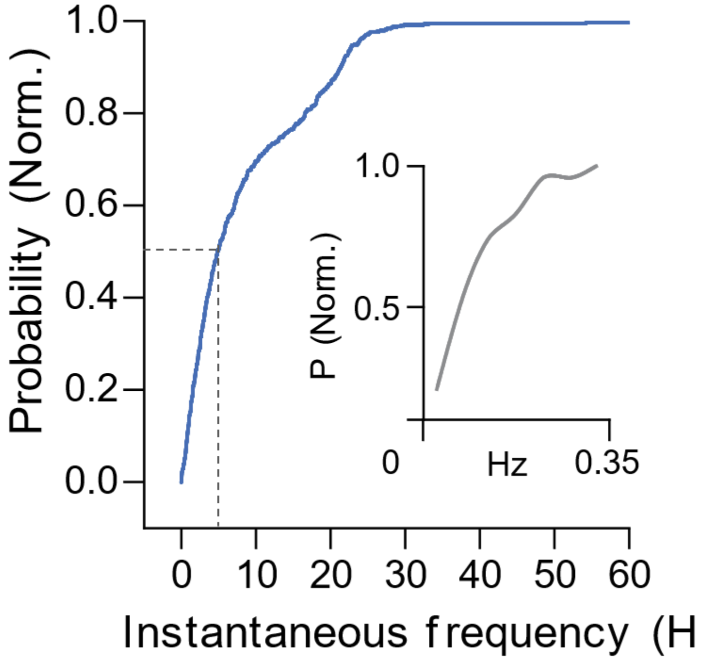

Figure S10: Cumulative distribution of frequency of action potentials in neurons cultured on top of glass coverslips (control, gray line, inset) and PDCBT-ITIC-P-*d*-L coated glass coverslips (blue line) after light exposure.

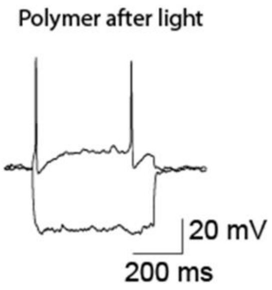

Figure S11: Voltage-time course traces, recorded from an individual neuron cultured on top of PDCBT-ITIC-P-*d*-L coated glass cover slips several minutes after light exposure.

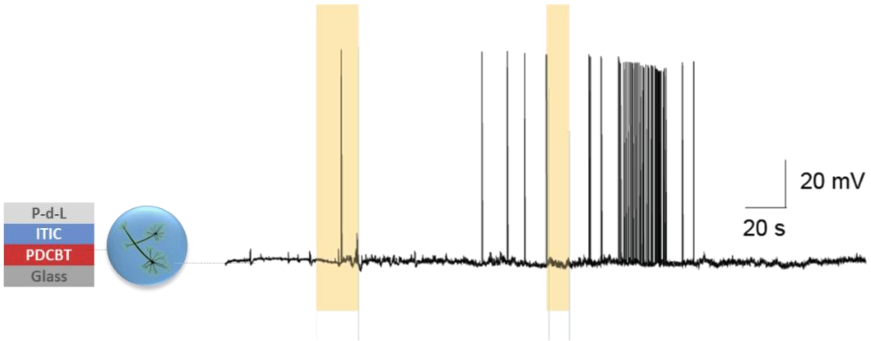

Figure S12: Membrane potential recordings of a mouse cortical neuron cultured on top of glass -PDCBT-ITIC-P-*d*-L coverslips and exposed to white light for several second. Action potentials are induced with significantly smaller initial depolarization of membrane potential.

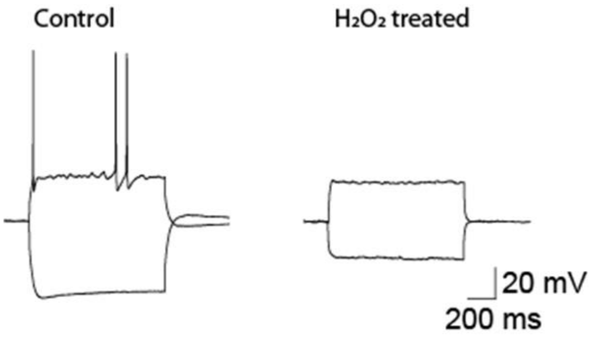

Figure S13: Representative voltage-time course traces, recorded from the same individual neuron cultured on glass – P-*d*-L substrates before, and after exposure in H_2O_2.

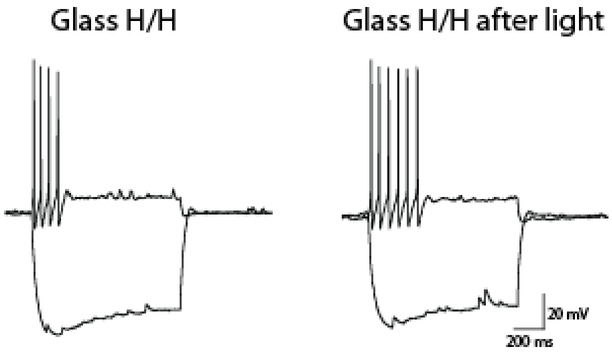

Figure S14: Representative voltage-time course traces, recorded from the same individual neuron cultured on half-coated glass – PDCBT-ITIC- P-*d*-L substrate before and after light irradiation.

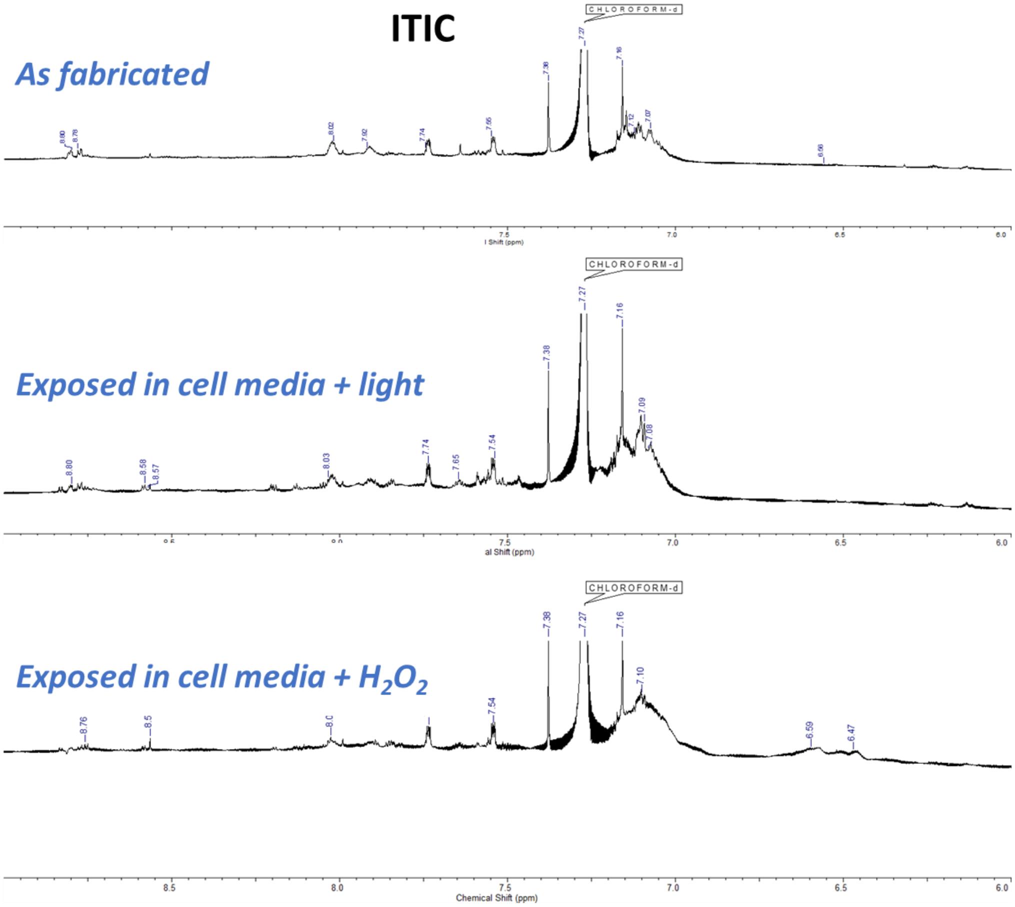

Figure S15: H-NMR spectra of ITIC as fabricated (top), after being exposed for 2 minutes in 40 mW/cm^2 white light irradiation in cell culture media (middle), and after being exposed in 500 um H_2O_2 added in cell culture media (bottom).

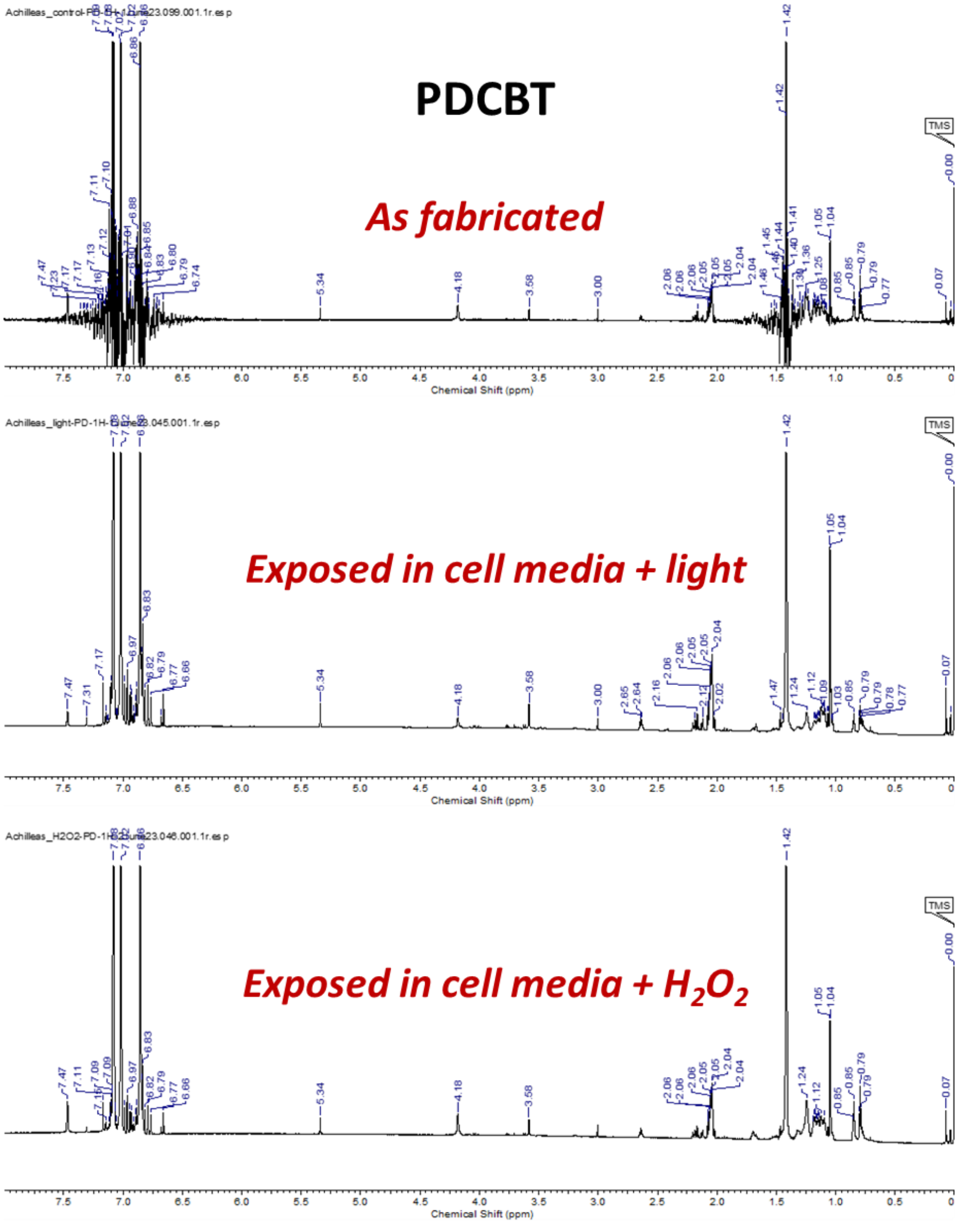

Figure S16: H-NMR spectra of PDCBT as fabricated (top), after being exposed for 2 minutes in 40 mW/cm^2 white light irradiation in cell culture media (middle), and after being exposed in 500 um H_2O_2 added in cell culture media (bottom).

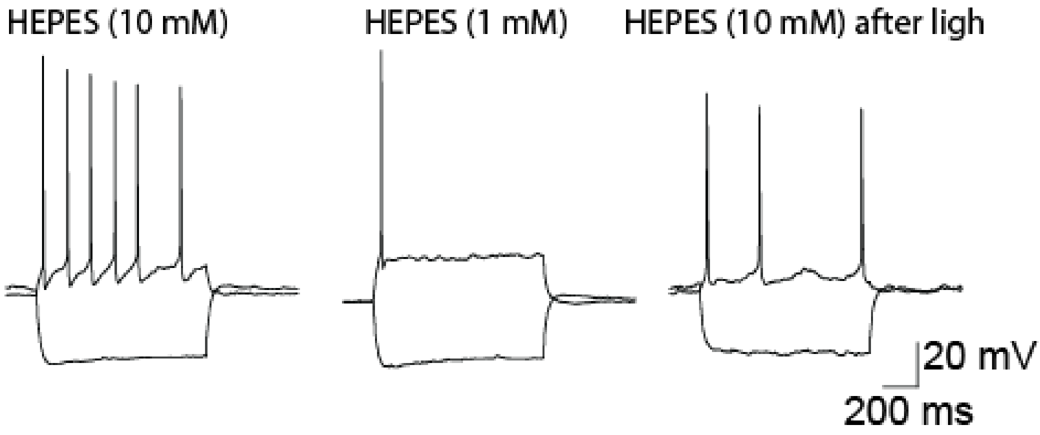

Figure S17: Representative voltage-time course traces, recorded from the same individual neuron cultured on fully coated PDCBT-ITIC- P-*d*-L glass cover slips in normal cell media containing 10 mM of HEPES (left), in cell media containing low concentration of HEPES (1 mM - middle) and again in normal media containing 10 mM of HEPES after being exposed in several consecutive light pulses for several seconds.

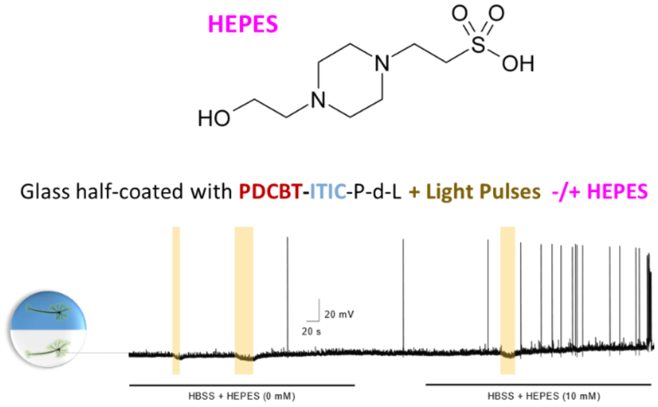

Figure S18: a) Representative membrane potential traces of a mouse cortical neuron cultured on glass cover slip half-coated with PDCBT-ITIC-P-*d*-L, in cell culture media with low HEPES concentration (1 mM) and in normal cell media containing 10 mM of HEPES. Action potential generation is induced only after light explosure in cell media containing 10 mM of HEPES.
